# Multi-Omics Analysis of the Effects of Sex on Flavor Formation in Xichuan Black-Boned Chicken Meat

**DOI:** 10.3390/ani16091287

**Published:** 2026-04-22

**Authors:** Li Zhou, Wenfei Dong, Zhiyuan Zhang, Xiangtao Kang, Yadong Tian, Xiaojun Liu, Ruili Han, Wenting Li, Donghua Li

**Affiliations:** College of Animal Science and Technology, Henan Agricultural University, Zhengzhou 450046, China; zhouli341125@126.com (L.Z.); 15103827042@163.com (W.D.); zhang90392023@163.com (Z.Z.); xtkang2001@263.net (X.K.); ydtian111@163.com (Y.T.); xjliu2008@hotmail.com (X.L.); rlhan@126.com (R.H.)

**Keywords:** chicken, sex, flavoromics, lipidomics, meat quality, multi-omics

## Abstract

Black-boned chicken is a native and valuable breed that is very important in the meat products of China. However, the molecular mechanisms underlying differences in muscle flavor between sexes remain unclear. In this study, 360-day-old male (BM, *n* = 6) and female (BF, *n* = 6) Xichuan black-boned chickens were used to screen differential lipids and differential flavor compounds in breast muscle tissue by lipidomics and flavoromics. This was followed by multivariate statistical analysis, functional enrichment and correlation network analysis of the differential lipids and flavor compounds obtained. Lipidomics identified 419 differential lipids associated with BM vs. BF, which were mainly enriched in glycerolipid metabolism and metabolic pathways. Flavoromics analysis identified 61 differential flavor compounds, and enrichment analysis showed that the terpenoid backbone biosynthesis pathway may be correlated with chicken muscle flavor formation. Correlation analysis revealed that triglyceride-type lipid molecules were closely related to the flavor compound 3-ethyl-2-methylheptane. These findings provide novel insights into the sex-related differences in the meat quality of Xichuan black-boned chickens, offering important data for their recognition and evaluation.

## Simple Summary

Flavor is a crucial indicator influencing consumers’ evaluation of chicken. Excellent flavor quality is an important factor for improving the added value of chicken products. However, the molecular mechanisms of flavor differences between male and female chickens remain largely unknown. In the current study, flavoromics and lipidomics techniques were employed to identify and analyze key lipid molecules and flavor compounds in the breast muscle of male and female Xichuan black-boned chickens. Overall, this study has enriched the understanding of the unique flavor formation in Xichuan black-boned chickens and provided an important theoretical basis for the molecular breeding of chickens with different sexes and the processing of flavored meat products.

## 1. Introduction

It is well known that meat is regarded as one of the primary sources of animal-sourced foodstuffs in the world [1]. Chicken is rich in nutrients and easily digestible, and contains abundant amino acids, trace elements, fatty acids, minerals, and vitamins [2]. There has been a rapid increase in the consumption of chicken meat over the past few decades, with demand expected to continue growing [3]. Moreover, chicken products are the most widely consumed animal-derived products across the world, since they are free from religious and cultural restrictions [4]. Meat quality is an extremely complex trait involving organoleptic characteristics, such as pH, color, tenderness, flavor, and juiciness, and is synthetically regulated by multiple factors [5]. Notably, sex is a primary influencing factor of meat quality when the genetic background and breeding approaches are consistent [6]. However, the specific regulatory mechanisms of muscle flavor formation in chickens between different sexes are not yet clear.

Xichuan black-boned chicken is a local chicken breed in China, which is mainly distributed in Xichuan County, Henan Province [7]. This breed is small in size and light in weight. Owing to its tenderness, succulence, and medicinal value, it is highly popular among local consumers [8]. The Xichuan black-boned chicken was officially recognized as a new breed in 2010. Its substantial meat production potential and quality advantages make it an important component of meat consumption in China [9]. Currently, there are few reports on the meat quality characteristics of this breed between different sexes [10]. This situation limits the full understanding of meat flavor in poultry and impedes the exploration of superior meat quality characteristics in poultry molecular breeding. Consequently, elucidating the molecular mechanisms underlying breast muscle flavor in male and female Xichuan black-boned chickens may be a research focus in poultry genetic breeding. Recent studies have widely applied multi-omics strategies to various meats [11]. Lipidomics, flavoromics, and metabolomics, as rapidly emerging technologies in recent years, provide a novel strategy for revealing the complex mechanisms of flavor formation through systematic analysis [12]. Among them, gas chromatography-mass spectrometry (GC–MS) is an analytical technique characterized by high sensitivity, excellent resolution and broad applicability [13]. It can systematically identify volatile compounds in muscle, thereby elucidating the chemical basis underlying flavor variations. Meanwhile, liquid chromatography-mass spectrometry (LC–MS) is utilized to accurately analyze the species and composition of lipid molecules in muscle [14]. This strategy elucidates the correlative function of lipid oxidation in flavor formation, thereby establishing a comprehensive molecular network underlying muscle flavor. Nevertheless, the specific divergences in lipid molecular composition, changes and flavor signatures across male and female chicken meat, along with their potential mechanisms, remain inadequately elucidated [15]. Consequently, studying the breast muscle in male and female Xichuan black-boned chickens can provide novel insights into meat quality enhancement and the elucidation of its regulatory mechanisms.

Therefore, the objectives of this study are to explore the differences in lipid and flavor profiles of the breast muscle between female and male Xichuan black-boned chickens based on lipidomics and flavoromics, and to investigate the relationships between lipids and flavor substances. The results from this study will improve our understanding of sex-related differences in chicken meat quality, providing a theoretical foundation for identifying key markers associated with chicken meat quality.

## 2. Materials and Methods

### 2.1. Animals and Sample Collection

All experimental procedures and animal care followed the protocols of the Animal Ethics Committee of Henan Agricultural University (S20190196). All Xichuan black-boned chickens were sourced from the Henan Agricultural University Breeding Factory in Henan Province, China. They were fed the same diet, and they could access food and water ad libitum under a standardized feeding and management regimen. A total of 6 male (BM) and 6 female (BF) Xichuan black-boned chickens of the same age were selected. All chickens were examined to make sure that they were in a healthy condition. At 360 days of age, the selected chickens were euthanized. Breast muscle samples from the chickens were collected immediately. Each sample was extracted from the same region of the right breast muscle using RNase-free scissors, with a moderate size. All breast muscle samples were carefully sealed and appropriately labeled. The muscle tissues were stored at −80 °C.

### 2.2. Lipid Isolation and Lipidomics Analysis

Lipids were extracted and weighed from the muscle samples of twelve Xichuan black-boned chickens following the method described by Li et al. [16] with minor modifications. In brief, 20 mg of each breast muscle sample was fully vortex mixed in a 1.5 mL capped Eppendorf tube using pre-cooled chloroform and methanol at a ratio of 2:1 (*v*/*v*). After being centrifuged at 12,000 rpm at 4 °C for 30 s, the samples were homogenized using steel balls for another 30 s. Subsequently, 900 μL of methyl tert-butyl methyl ether was added, and the mixture was vortexed thoroughly. After vortexing the mixture for 5 min, 200 μL of deionized water was supplemented. The upper organic phase was collected after centrifugal treatment at 12,000 rpm at 4 °C for 10 min. Subsequently, the organic phase was collected and dried under vacuum conditions. The dried lipid extracts were reconstituted in a lipid solution (acetonitrile:isopropanol = 1:1, *v*/*v*) and stored at −20 °C.

To assess and analyze stability, a quality control (QC) sample was prepared by pooling equal aliquots of all samples, and one QC sample was inserted after every six samples. Chromatographic separation was performed on an ultra-high-performance liquid chromatography (UHPLC) system (ExionLC™ AD, SCIEX, Marlborough, MA, USA) coupled with a C30 chromatographic column. The ultra-high-performance liquid chromatography was performed under the following analytical conditions: The mobile phase consisted of phase A and phase B. Phase A was acetonitrile/water (60/40, *v*/*v*) supplemented with 10 mmol/L ammonium formate and 0.1% formic acid, while phase B was acetonitrile/isopropanol (10/90, *v*/*v*) supplemented with 10 mmol/L ammonium formate and 0.1% formic acid. The gradient elution program was set as follows: from 0 to 2 min, phase B increased gradually from 20% to 30%; 4–9 min, phase B increased gradually from 60% to 85%; 14–15.5 min, phase B remained between 90% and 95%; 17.3 min, phase B remained at 95%, 17.5–20 min, phase B returned to 20%. The flow velocity was 0.35 mL/min, the column temperature was maintained at 45 °C and the injection volume was 2 μL.

The samples were analyzed by UHPLC coupled with tandem mass spectrometry on a QTRAP^®^ 6500^+^ system (SCIEX, MA, USA). Mass spectrometry was conducted under the following conditions: electrospray ion source (ESI) temperature—500 °C; positive mode spray voltage—5.5 kV; negative mode spray voltage—4.5 kV; curtain gas (CUR), 35 psi; and ion sources—gas 1 (GS1), 45 psi, gas 2 (GS2), 55 psi.

Positive and negative ionization modes were used for analysis. Multiple reaction monitoring (MRM) was utilized for triple quadrupole scanning with collision gas. The declustering potential (DP) and collision energy (CE) after parameter optimization were applied to establish individual MRM transitions. The acquired raw data were analyzed using analyst 1.6.3 software (SCIEX, MA, USA). MultiQuant software (version 3.0.2; AB Sciex, Concord, ON, Canada) was utilized for peak extraction, identification, alignment, integration, and retention time (RT) correction. The lipids were identified by matching against the self-built database MWDB (MetWare database) based on RT, molecular weight of parent ion, and characteristic fragment ions. The lipid molecules were quantified using MRM mode.

Principal Component Analysis (PCA) was employed to observe sample classification or discrimination in the multivariate space. To evaluate the sample classification performance, a partial least squares-discriminant analysis (PLS-DA) model was performed by implementing a dummy Y-variable for each group. Orthogonal partial least squares-discriminant analysis (OPLS-DA), which separates systematic variation into predictive (X matrix) and orthogonal (Y matrix) components, was further employed to facilitate the interpretation of group separation. The classificatory and predictive abilities were evaluated on the basis of the cumulative parameters R^2^Y and Q^2^Y. Lipid molecules that were recognized as statistically significant were those with Variable Importance in the Projection (VIP) scores > 1 and false discovery rate (FDR) < 0.05. The VIP scores were extracted from the OPLS-DA model, while the *p*-values were calculated by Student’s *t*-test. Kyoto Encyclopedia of Genes and Genomes (KEGG) pathways were considered significantly enriched when the FDR-corrected *p*-value < 0.05.

### 2.3. Flavor Compound Isolation and Flavoromics Analysis

Flavor compounds were isolated based on the approach reported by He et al. [17] with slight modifications. Briefly, 2.0 g of samples were incubated with 10 μL of the internal standard solution (1-Hexyl-d13) at 60 °C for 15 min. The SPME fiber (Supelco, Bellefonte, PA, USA) was preconditioned at 250 °C for 10 min prior to the isolation procedure. Then, the samples were extracted using the fiber at 60 °C for 20 min. Desorption of the fiber was performed in the GC inlet at 250 °C for 10 min. The sample was immediately stored at −80 °C. All flavor compound extracts were analyzed by GC-MS. Each sample was analyzed in triplicate. The analysis was carried out using an Agilent 8890 gas chromatograph (Agilent Technologies, Santa Clara, CA, USA) equipped with a 7000D mass spectrometer (Agilent Technologies, USA). A DB-5MS capillary column (Agilent, USA) was used for chromatographic separation. High-purity helium (>99.999%) was used as the carrier gas. The flow rate was maintained at a constant 1.2 mL/min. The column temperature program was programmed as follows: initial temperature was held at 40 °C for 5 min, subsequently ramped at 10 °C/min to 100 °C, followed by a ramp at 7 °C/min to 180 °C and kept for 10 min, and finally increased at 25 °C/min to 280 °C and maintained for 5 min.

Mass spectrometry parameters were established as follows: electron impact energy was 70 eV. Quadrupole temperature, ion source temperature, and transfer line temperature were held at 150 °C, 230 °C, and 250 °C, respectively. Mass spectrometry data were collected in full-scan mode over the range *m*/*z* 20–400 amu. Full-scan mass spectrometry data were acquired over the range of *m*/*z* 20–400 amu, with a 3.5 min solvent delay applied.

The data on flavor compounds were analyzed using MassHunter (version 8.0) software. Volatile compounds were identified by matching mass spectra against the NIST 2017 (Gaithersburg, MD, USA) database, linear retention index (RI), and search software. The detection rate of flavor compounds in each sample was calculated, and the flavor compounds with a detection rate > 50% were retained. The sensory odor traits of Xichuan black-boned chickens were analyzed in accordance with the online database (http://www.thegoodscentscompany.com, accessed on 18 April 2023). Additionally, flavor compounds in the muscle of black-boned chickens were characterized in terms of their types and contents using the PubChem and Classyfire databases [18]. The coefficient variation (CV) values of peak areas for flavor compounds in each sample were determined. Only compounds with CV < 30% were kept for further analysis. The data were subsequently subjected to PCA and OPLS-DA using the R package (version 3.5.1) and MetaboAnalystR (version 1.0.1), respectively. Differential flavor compounds were initially selected using VIP > 1.0, and Fold Change ≥ 1.5 or Fold Change ≤ 0.67. Similarly, the functional roles of the differential flavor compounds were analyzed via KEGG pathways. Pathways with a *p*-value < 0.05 were considered significantly enriched, and the *p*-value was adjusted using the Q-value.

### 2.4. Correlation Analysis of Differential Lipids and Flavor Compounds

To investigate the influence of differential flavor compounds and lipids on chicken flavor, the present work performed Spearman′s correlation analysis on the flavoromics and lipidomics data through the R base package (version 4.4.0). In the joint analysis of multi-omics, the strong correlations between differential flavor compounds and differential lipids were determined based on *p*-value < 0.05 and |r| > 0.5, thereby screening the key flavor compounds and lipids that contribute to chicken flavor. Furthermore, network diagrams were established to visualize the interactions between these lipid molecules and flavor compounds.

### 2.5. Data Analysis

Student’s *t*-test was used to analyze significant differences between groups. The one-way ANOVA program in SPSS statistics (version 26; IBM) was used for analysis of variance. The results were expressed as mean ± standard deviation (SD). Graphs were generated with GraphPad Prism 8.0 software. Statistical significance was denoted as *p*-value < 0.05.

## 3. Results

### 3.1. Lipid Molecular Characteristics in Lipidomics

**Figure 1:**
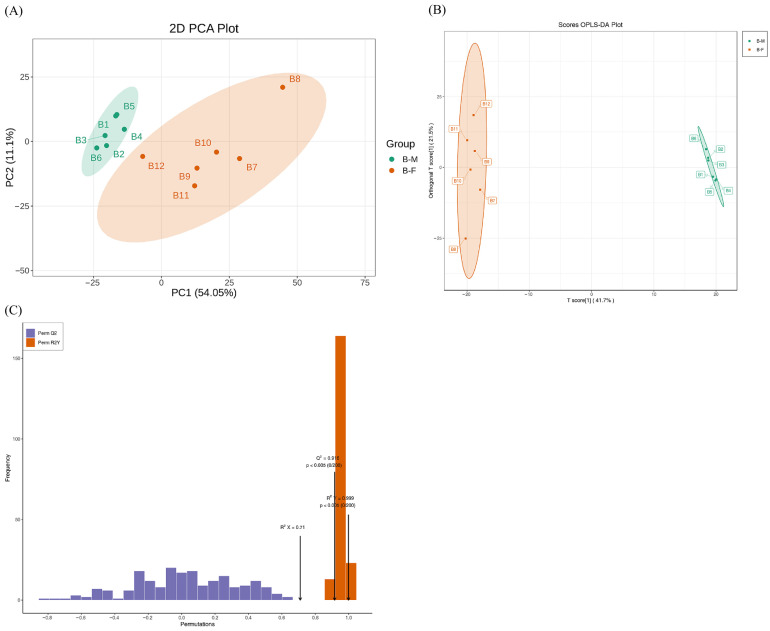
Multivariate statistical analysis of lipidomics. (**A**) PCA of the lipid molecules. B1–B6 represent samples of the BM group in lipidomics, and B7–B12 represent samples of the BF group in lipidomics. PC1 shows principal component 1, and PC2 shows principal component 2. (**B**) OPLS-DA score plots of the lipid molecules. B1–B6 show samples of the BM group; B7–B12 show samples of the BF group. (**C**) OPLS-DA validation plot of the lipid molecules (R^2^X = 0.71, R^2^Y = 0.999, and Q^2^ = 0.916).

Lipids were extracted from 12 Xichuan black-boned chickens using the LC-MS/MS platform. To investigate the lipid profiles of the BM and BF groups, multivariate statistical analysis was performed to explore the differences in chicken meat samples. The 2D PCA score plot revealed that PC1 and PC2 explain 54.05% and 11.1% of the total variance, respectively (combined explained variance: 65.15%), and the close clustering within each group verifies reliable reproducibility of both the BM and BF groups (Figure 1A). Subsequently, an OPLS-DA score plot was used to differentiate samples between the B-M and B-F groups (Figure 1B). The model revealed that T score and orthogonal T score explained 41.7% and 21.5% of the total variance, respectively, with a combined explained variance of 63.2%, complete separation of B-M and B-F samples along the T score axis with no overlap, and dense clustering within each group verifying satisfactory intragroup reproducibility. The OPLS-DA score plot showed that the samples were distinctly separated, suggesting a significant difference between the two groups. The permutation test for the OPLS-DA model (*n* = 200) showed that R^2^Y was 0.999, R^2^X was 0.71, and Q^2^ was 0.916. Therefore, this original model possessed better accuracy and robustness (Figure 1C).

### 3.2. Identification of Differential Lipids in Lipidomics

**Figure 2:**
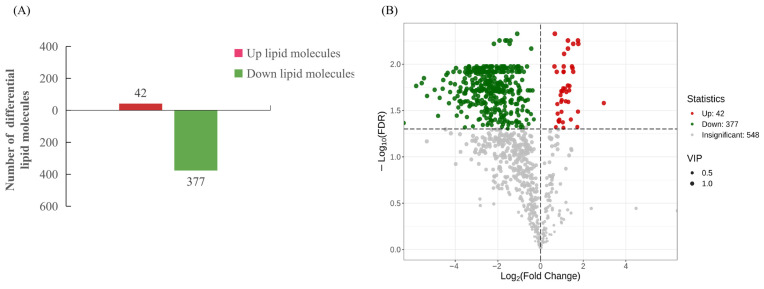
Identification of differential lipids in lipidomics. (**A**) Statistical analysis of the number of differential lipids. The green bar depicts down-regulated lipid molecules, while the red bar depicts up-regulated lipid molecules. (**B**) Volcano plot of lipid molecules. The horizontal axis represents the log2 Fold Change in lipid molecules, and the vertical axis represents −log10 *p*-value. Green dots indicate down-regulated lipid molecules, and red dots indicate up-regulated lipid molecules.

Subsequently, VIP scores were derived from the OPLS-DA model. To further investigate lipid differences between the BM and BF groups, based on the criteria (VIP > 1 and FDR < 0.05), a total of 419 differential lipid molecules were identified in this study (Supplementary Table S1). Compared to the BF group, 42 lipid molecules were up-regulated in the BM group, whereas 377 lipid molecules were down-regulated (Figure 2A). The volcano plots were generated to visualize 377 down-regulated and 42 up-regulated lipid molecules between male and female Xichuan black-boned chickens (Figure 2B). Collectively, the main classes of the above differential lipid molecules were phosphatidylethanolamines (PE), phosphatidylcholines (PC), and triglycerides (TG).

### 3.3. Total Lipid Difference Analysis in Lipidomics

**Figure 3:**
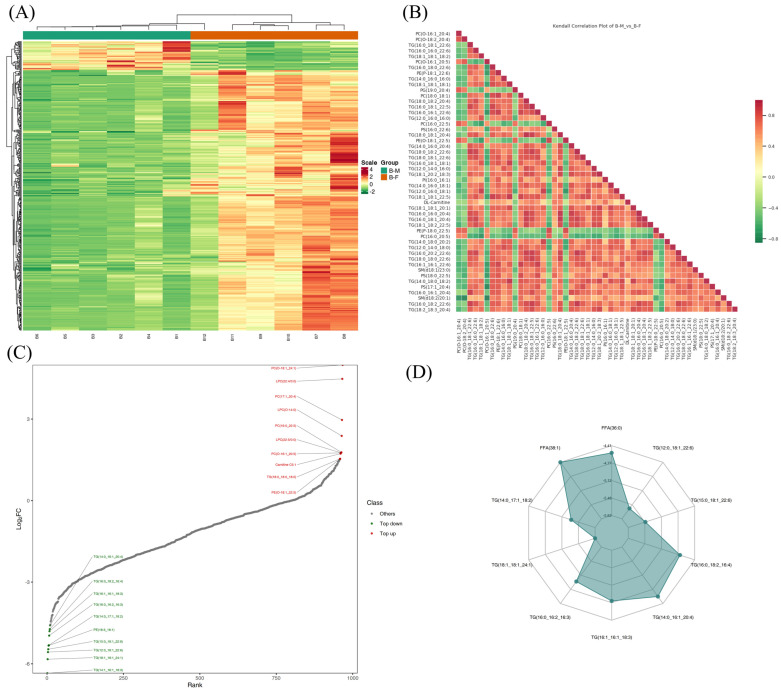
Total lipid difference analysis of lipidomics. (**A**) Heatmap of differential lipid molecules. The horizontal axis represents the sample name, and the vertical axis represents lipid molecule information. B1–B6 indicate samples of the BM group in lipidomics, and B7–B12 indicate samples of the BF group in lipidomics. (**B**) Correlation analysis for differential lipid molecules (top 50 VIP scores). Red denotes positive correlation, and green denotes negative correlation. (**C**) Dynamic distribution map of lipid molecule content differences. The horizontal axis in the figure represents the cumulative amount of the lipid molecules, and the vertical axis represents log2 Fold Change. Green represents the top 10 down-regulated lipid molecules, and red represents the top 10 up-regulated lipid molecules. (**D**) Radarchart of differential lipid molecules. The grid lines correspond to the magnitude of the Fold Change value, and the green shadow is composed of the Fold Change value corresponding to each lipid.

Heatmap cluster analysis indicated high intra-group consistency and significant inter-group differences, enabling effective differentiation between male and female Xichuan black-boned chickens (Figure 3A). The color scale of the heatmap showed normalized expression levels from −2 to 4. Higher content than the average was labeled as red, and lower content was labeled as green. Pearson’s correlation analysis was conducted for the significantly differential lipids, and the top 50 variables with the highest VIP scores were visualized, demonstrating that positively correlated lipids were more abundant than negatively correlated ones (Figure 3B). The top 10 up-regulated and top 10 down-regulated lipid molecules were distributed in a dynamic manner, as shown in Figure 3C. Based on the screening criteria, the top 10 lipid molecules with the largest FC values were selected for Radarchart analysis, revealing that these lipids were mainly TG types (Figure 3D).

### 3.4. Lipid Molecules Pathway Enrichment Analysis in Lipidomics

**Figure 4:**
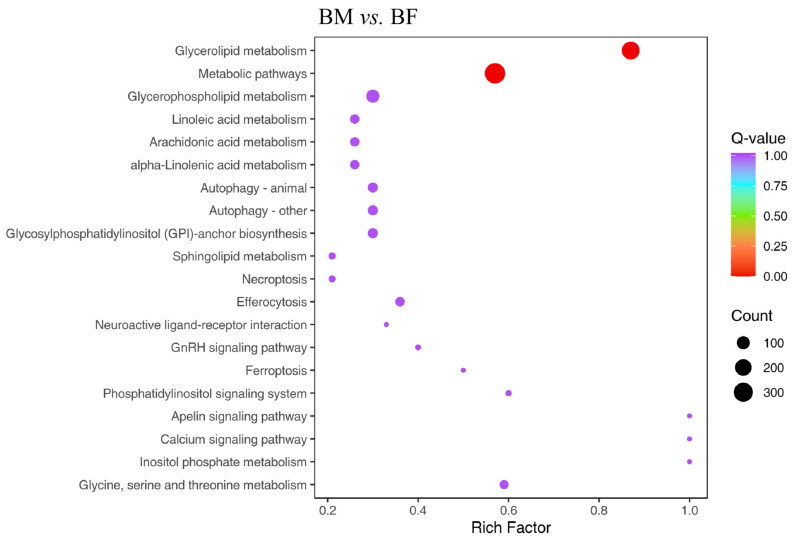
KEGG enrichment analysis of differential lipid molecules. The horizontal axis represents the enrichment factor of each pathway, and the vertical axis displays the corresponding pathway names. The circle color reflects the *p*-value, and the circle size represents the number of differential lipid molecules enriched in the corresponding pathway.

To further understand the potential biological significance of the differential lipid molecules, systematic pathway enrichment analysis was performed using the KEGG database (Figure 4). These pathways included glycerolipid metabolism and metabolic pathways. These results provide new perspectives on the biochemical mechanisms governing lipid composition in Xichuan black-boned chicken muscle.

### 3.5. Flavor Characteristics in Flavoromics

**Figure 5:**
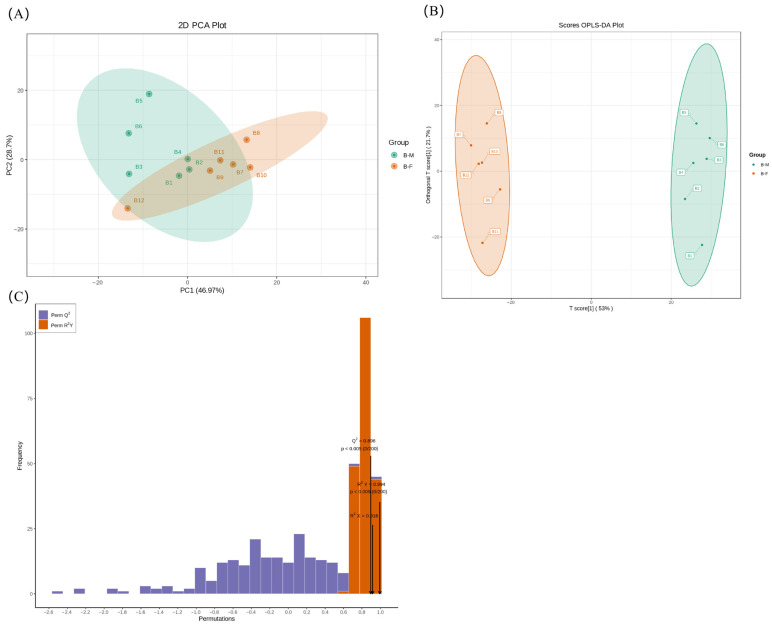
Multivariate statistical analysis of flavoromics. (**A**) PCA of flavor compounds. B1–B6 represent samples of the BM group in flavoromics; B7–B12 represent samples of the BF group in flavoromics. PC1 shows principal component 1; PC2 shows principal component 2. (**B**) OPLS-DA score plot of the flavoromics. (**C**) OPLS-DA validation plots of the flavoromics (R^2^X = 0.916, R^2^Y = 0.994, and Q^2^ = 0.896).

Besides lipid molecules, flavor compounds also significantly influence meat flavor. Accordingly, flavor compounds from male and female Xichuan black-boned chickens were analyzed to further explore the flavor differences between them. In this study, the flavor compound in Xichuan black-boned chicken was identified by GC-MS/MS. The PCA results indicated that the cumulative variance explained by the first two components was 75.67% (PC1 46.97% and PC2 28.7%), as shown in Figure 5A. To maximize sex discrimination, a supervised OPLS-DA score plot was generated to effectively differentiate male and female Xichuan black-boned chickens (Figure 5B). The OPLS-DA revealed that the T score and the orthogonal T score accounted for 53% and 21.7% of the total variance, respectively, representing a total explained variance of 74.7%, with complete separation of B-M and B-F samples along the T score axis and tight intra-group clustering verifying reliable reproducibility. Furthermore, validation of a 200-permutation test revealed that the OPLS-DA model yielded R^2^Y = 0.994, R^2^X = 0.916, and Q^2^ = 0.896, confirming its robustness and showing no overfitting (Figure 5C).

### 3.6. Identification of Differential Flavor Compounds in Flavoromics

**Figure 6:**
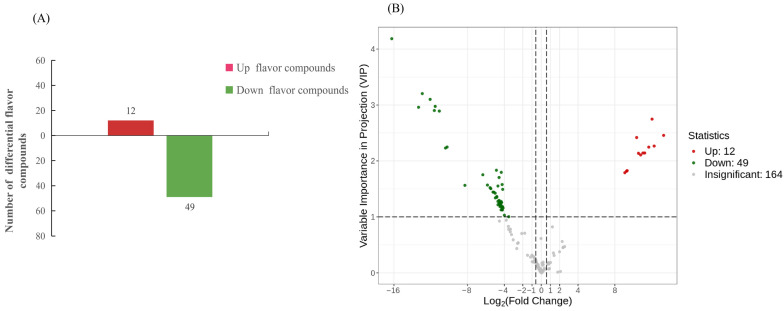
Identification of differential flavor compounds in flavoromics. (**A**) Statistical analysis of the number of differential flavor compounds. The green bar shows down-regulated flavor compounds, while the red bar shows up-regulated flavor compounds. (**B**) Volcano plot of flavor compounds. The horizontal axis indicates the log2 Fold Change in flavor compounds, and the vertical axis indicates −log10 *p*-value. Green and red dots represent down-regulated and up-regulated differential flavor compounds, respectively.

According to the screening criteria for flavor compounds, a total of 61 differential flavor compounds were identified in the BM vs. BF (Supplementary Table S1), among which 12 were up-regulated, and 49 were down-regulated (Figure 6A). Moreover, a volcano plot was drawn to display the overall distribution of 12 up-regulated and 49 down-regulated differential flavor compounds (Figure 6B). Overall, the main classes of identified flavor compounds were hydrocarbons, aldehydes, and esters.

### 3.7. Total Lipid Difference Analysis in Flavoromics

**Figure 7:**
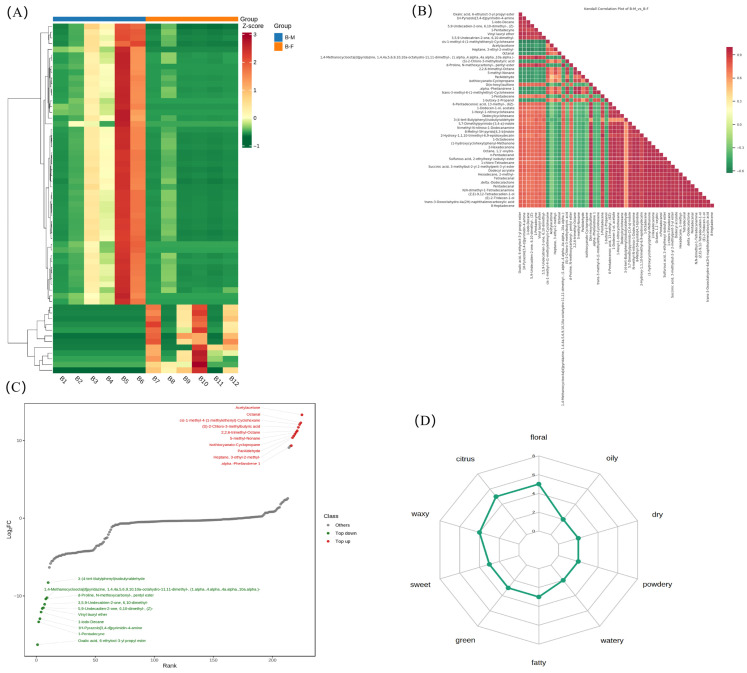
Total flavor compounds of flavoromics. (**A**) Heatmap of differential flavor compounds. Horizontal axis indicates sample names; vertical axis indicates flavor compound information. B1–B6 represent samples of the BM group in flavoromics; B7–B12 represent samples of the BF group in flavoromics. (**B**) Correlation analysis for differential flavor compounds (top 50 VIP scores). Red shows positive correlation; green shows negative correlation. (**C**) Dynamic distribution map of flavor compounds content differences. The horizontal axis in the figure shows the cumulative amount of the substance, and the vertical axis shows log2 Fold Change. Green and red represent the top 10 down-regulated and top 10 up-regulated flavor compounds, respectively. (**D**) Radarchart of differential flavor compounds. The grid lines correspond to the magnitude of the Fold Change value, and the green shadow is composed of the Fold Change value corresponding to each flavor compound.

To facilitate a more thorough investigation of the effects of differential flavor compounds on clustering, a clustering heatmap was produced. The analysis showed a consistent overall trend in the content of all flavor components in BM vs. BF, with minor variations in the content of flavor components (Figure 7A). The Z-score range of the heatmap was −1 to 3. Higher content than the average was labeled as red, and lower content was labeled as green. This further demonstrates that the flavor characteristics of breast muscles in male and female Xichuan black-boned chickens are significantly different, but also have similarities. Pearson correlation analysis was performed on the differential flavor compounds screened according to the established criteria. The results showed that the top 50 flavor compounds with the highest VIP scores were mostly positively correlated (Figure 7B). Furthermore, the differences in flavor compound content were ranked from small to large according to FC values, and a dynamic distribution map of flavor compound content differences was drawn, with the top 10 up-regulated and top 10 down-regulated flavor compounds marked (Figure 7C). Sensory characteristics of the flavor compounds in breast muscle from male and female Xichuan black-boned chickens were predicted using the PubChem and Classyfire online databases, and the results showed that they may have higher sensory scores for floral and citrus aromas (Figure 7D).

### 3.8. Flavor Compounds Pathway Enrichment Analysis in Flavoromics

**Figure 8:**
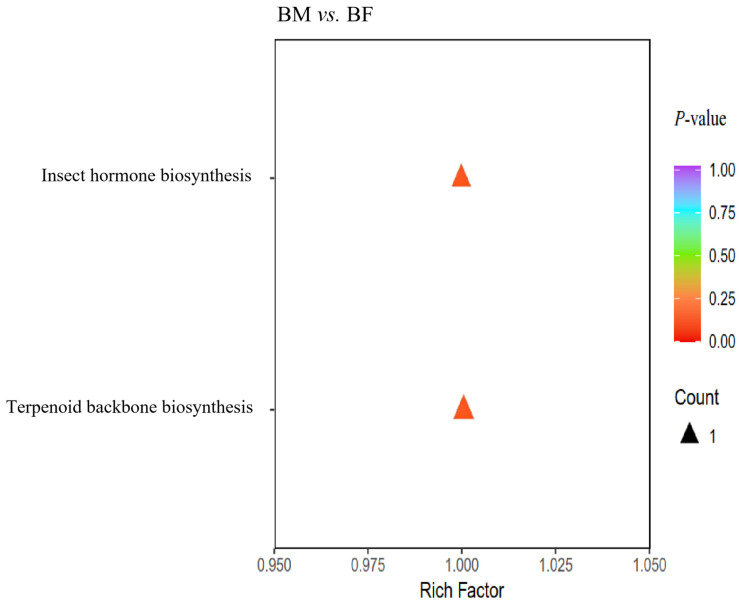
KEGG enrichment analysis of differential flavor compounds. The horizontal axis represents the enrichment factor of each pathway, and the vertical axis shows the pathway name (*p*-value). The triangle color reflects the *p*-value, and the triangle size represents the number of differential flavor compounds enriched in the corresponding pathway.

To better understand the potential biological significance of the differential flavor compounds, systematic pathway enrichment analysis was performed using the KEGG database (Figure 8). The results showed that the terpenoid backbone biosynthesis signaling pathway may directly or indirectly exert potential effects on the flavor compounds in the muscle of male and female Xichuan black-boned chickens.

### 3.9. Correlation Analysis Between Differential Lipid Molecules and Flavor Compounds

**Figure 9:**
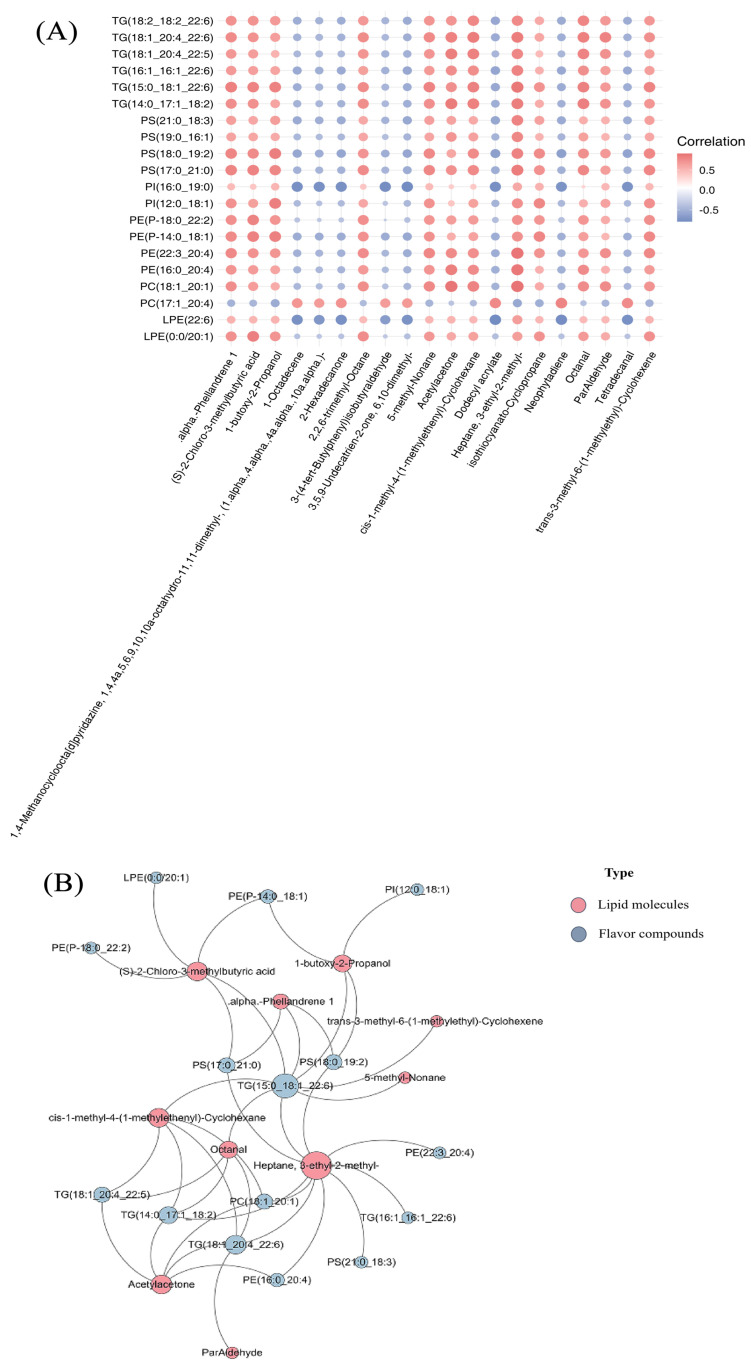
Correlation network analysis of lipidomics and flavoromics. (**A**) Matrix of top 20 differential flavor compounds and lipid molecules. The horizontal axis shows differential lipid molecules, and the vertical axis shows differential flavor compounds. Red indicates positive correlations, while blue represents negative correlations. (**B**) Correlation network analysis between differential lipid molecules and flavor compounds (|r| > 0.5 and *p* < 0.05). The size of the nodes represents the number of substances that are significantly correlated with the substance in question. Pink circles show flavor compounds, and blue circles represent differential lipid molecules.

To further explore the interaction between the above differential lipid molecules and flavor compounds, the Pearson correlation coefficients were calculated. The matrix diagram analysis results revealed significant positive or negative correlations between the top 20 differential lipid molecules and the top 20 differential flavor compounds (Figure 9A). In this study, Pearson′s correlations of differential lipid molecules and differential flavor compounds in the Xichuan black-boned chickens were also calculated, and only strong (|r| > 0.5) and significant (*p*-value < 0.05) correlations were retained, as shown in Figure 9B. Compared with the BM group, the BF group showed strong intramolecular correlations between the lipid subclass molecule TG and the flavor compound 3-ethyl-2-methylheptane.

## 4. Discussion

Lipid molecules are essential components of meat and important functional substances in organisms [19]. To further study the lipid changes in the Xichuan black-boned chicken meat, we performed a widely targeted lipidomics analysis of breast muscle based on the LC-MS/MS system. The PCA indicated that the lipid profiles in male and female Xichuan black-boned chickens were clearly distinguished. To further expand the differences between the groups, it was beneficial to find the differential lipids between the BM and BF groups. We established an OPLS-DA model to further analyze the breast meat samples. The OPLS-DA results showed that there was dispersion between the principal components of BM and BF. This model showed good predictive performance based on 200 permutations of the experimental results [20]. Studies have demonstrated that sex impacts meat quality and lipid composition in poultry [21]. Consistent with these results, our findings indicate that sex differences exert an impact on muscle lipid molecules. It has been reported that TG is mainly stored in lipid droplets in muscle [22]. TG is an energy supply and dynamic fat-storage depot and plays an important regulatory role in the metabolism of the body [23]. Phospholipids mainly consist of phosphatidylcholine (PC), phosphatidylethanolamine (PE) and sphingomyelin [24]. Phospholipids, particularly PE and PC, contain unsaturated fatty acyl chains that are highly susceptible to oxidation, decomposing earlier and more rapidly and thereby initiating the lipid oxidation cascade [25]. Previous studies have shown that phospholipids are the major contributors to the increase in free fatty acids during the formation of the characteristic aroma of Nanjing dry-cured duck meat [26]. Jin et al. (2024) employed lipidomics to analyze the livers of giant salamanders from different age and sex groups, reporting that TG and PC were the main identified lipids, which was aligned with our findings [27]. These differential lipid molecules of breast muscles in male and female Xichuan black-boned chickens were enriched in the lipid metabolism-related pathways (e.g., glycerolipid metabolism and metabolic pathways). Glycerolipid metabolism was the key regulatory pathway in Tibetan chicken [28]. Metabolic pathways are known to be used for the synthesis or decomposition of substances, the conversion of energy, and the maintenance of the organism’s life activities [29]. The metabolic pathway plays an essential role in skeletal muscle development, which explains why this pathway is found in the muscle lipidome [30]. Overall, changes in lipid revealed the effect of sex on chicken meat quality and revealed significantly different sex-related lipid pathways, including glycerolipid metabolism and threonine metabolism, and metabolic pathways.

Beyond lipid composition, we also investigated the effects of heating on the flavor composition in Xichuan black-boned chicken meat using flavoromics, which has the advantages of good reproducibility and high quantitative accuracy. The PCA and OPLS-DA score plots of flavor substances in Xichuan black-boned chicken breast muscle in different sexes showed that there were obvious differences in volatile flavor compounds between male and female Xichuan black-boned chicken breast muscle, and the sex of Xichuan black-boned chicken can greatly affect the volatile flavor compounds in Xichuan black-boned chicken breast muscle. The volatile flavor compounds in the muscles of livestock and poultry are mainly determined by the type and amount of lipids [31]. Lipolysis of adipose tissue is a catabolic process that breaks down triglycerides, stored fatty acids and glycerol in fat cells [32]. Via free radical chain reactions, unsaturated fatty acids act as substrates to form lipid hydroperoxides during lipid autoxidation [33]. These lipid hydroperoxides are further decomposed into flavor compounds including alcohols, aldehydes, hydrocarbons, ketones and acids [34]. At present, it is generally believed that these volatile flavor compounds are the main source of the unique flavor of muscle [35]. In this study, the majority of volatile flavor compounds were hydrocarbons, aldehydes and esters, a result that aligns with the existing literature [36,37]. As for hydrocarbons, aliphatic hydrocarbons arise from lipid oxidation, while aromatic hydrocarbons are generated through the oxidation of branched aromatic acids [38]. Characterized by fatty aromas, most aldehydes serve as critical intermediates in Maillard and lipid oxidation reactions and participate in reactions between amino acids and carbonyl groups [39]. The primary sources of esters are esterification and transesterification reactions [40]. Therefore, it was speculated that these flavor compounds might directly or indirectly correlate with the flavor formation of chicken meat. Functional enrichment analysis revealed that these differentially expressed lipid metabolism-related flavor compounds were predominantly enriched in biological processes such as terpenoid backbone biosynthesis. Previous studies have shown that the terpenoid backbone biosynthesis is a key signaling pathway for intramuscular fat deposition in broiler chickens [41]. Similarly, it has been reported that most of the differential metabolites in the breast muscle of Muchuan black-boned chickens are significantly enriched in the terpenoid backbone biosynthesis pathway [42]. Interestingly, terpenoid backbone biosynthesis is one of the essential pathways for the biosynthesis of flavor-related compounds [43]. It is inferred that the above signaling pathways may be directly or indirectly associated with biological processes related to chicken muscle metabolism.

Presently, an increasing number of studies are striving to pinpoint flavor components associated with differential lipids with more precision and elucidate the molecular mechanisms underlying flavor formation using integrated lipidomics and flavoromics analysis [44]. To explore the underlying mechanisms of chicken muscle flavor, the correlation analysis of differential lipid molecules and flavor compounds was performed. Correlation analysis further confirmed a strong positive correlation between the flavor compound 3-ethyl-2-methylheptane and the lipid molecule TG. This aligns with previous lipidomics results that TG lipids serve as primary markers for distinguishing lipid composition differences in the breast muscles of poultry [45]. However, our study also has certain limitations, including the limited number of lipidomics and flavoromics samples, which severely hindered the efficient identification of additional lipid molecules and flavor compounds. Additionally, experimental validation of the correlation between differential lipid molecules and flavor compounds is lacking, and this will be further investigated in future work.

## 5. Conclusions

This is the first application of multi-omics to describe the flavor characteristics of Xichuan black-boned chicken meat at different sexes. A total of 419 lipid metabolites were identified in Xichuan black-boned chicken muscles of different sexes. TG, PE, and PC were the most abundant types of lipids. Meantime, a total of 61 flavor compounds were detected in Xichuan black-boned chicken muscles based on flavoromics. Hydrocarbons, aldehydes, and esters were the predominant categories of flavor compounds. It identified key flavor compounds—primarily involved in the terpenoid backbone biosynthesis pathway—that may serve as biomarkers for chicken muscle flavor formation, particularly 3-ethyl-2-methylheptane. These findings not only enrich our understanding of the lipid and volatile profiles in Xichuan black-boned chicken muscles in relation to sex, but also offer valuable insights for sex identification.

## Data Availability

The original contributions presented in this study are included in the article/Supplementary Material.

## Supplementary Materials

The following supporting information can be downloaded at: https://www.mdpi.com/article/10.3390/ani16091287/s1, Table S1: Differentially expressed lipid molecules; Table S2: Differentially expressed flavor compounds.
